# Fragments Along the Way: Minimalism as an Account of Some Stages in First Language Acquisition

**DOI:** 10.3389/fpsyg.2020.00584

**Published:** 2020-05-08

**Authors:** Helen Goodluck, Nina Kazanina

**Affiliations:** ^1^Department of Language and Linguistic Science, University of York, York, United Kingdom; ^2^School of Psychological Science, University of Bristol, Bristol, United Kingdom; ^3^Centre for Cognition and Decision Making, Institute for Cognitive Neuroscience, HSE University, Moscow, Russia

**Keywords:** first language acquisition, minimalism, performance mechanisms, merge, pronoun interpretation, questions

## Abstract

We discuss two instances in which the minimalist model of syntax offers a potential account of children's linguistic behavior: the Merge analysis of phrase structure and the analysis of pronominal structures and other long distance dependencies. In each case, we need to understand the relationship between performance mechanisms (the mechanisms for language production and comprehension) and the syntax on which these mechanisms draw.

In this article we will explore some of the potential that comes out of Minimalist syntax for an account of stages in language acquisition, focussing on the early emergence of word order, and the role of interface conditions in explaining children's behavior. Our discussion does not aim to be a comprehensive account of language acquisition in a Minimalist framework—such an account would require far more research, which is (to our knowledge) yet to be done. However, we can point to a common thread in the examples we discuss: In each case, we need to understand the relationship between performance mechanisms (the mechanisms for language production and comprehension) and the syntax on which these mechanisms draw.

## Merge in Syntactic Theory

In the work of Chomsky (1995, and subsequent publications), the operation of Merge is fundamental to structure building. It is an operation that combines two syntactic units into a constituent. Asymmetric Merge determines which of the two elements is the head of the unit: in languages such as English, it is the left element that is the head and determines the category label of the constituent; in language such as Japanese, it is the right element that is the head and determines the category label.

### Merge as an Account of Early Stages in Language Development

Braine (1963) provides an early report of the young children's attempts to combine words. Braine gives evidence of the three children (Gregory, Andrew and Steven) he studied producing “Pivot” and “Open” classes of words^1^. Pivots are words such as *allgone, byebye*, and *see* that occur in the majority of word combinations, and to which other words from the open class are attached, e.g., *allgone shoe* and *allgone egg*, or *byebye plane* and *byebye man*. The data from the three children reported in Braine's article is given in Table 1, in abbreviated form. Braine observed a period of about 4 months (from the first occurrence of two word utterances at approximately 19 months) in which the Pivot-before-Open (hereafter Pivot-Open) pattern predominates for Gregory and Andrew; Steven also had a Pivot-Open pattern in examples tape-recorded in the fourth and fifth months after the first occurrence of two word utterances.

**Table 1:** Analysis of speech data from three children (Braine, 1963).

**Pivot-Open**	**Open-Pivot**	**Other**
**Gregory (age 19–22 months)**		
BYEBYE [31] *byebye plane, byebye man, byebye hot*,. SEE [14] *see boy, see sock, see hot, …* ALLGONE [5] *allgone shoe, allgone vitamins, allgone egg, allgone lettuce, allgone watch* MY [3] *my mummy, my daddy, my milk* BIG [3] *big boss, big boat, big bus* PRETTY [2] *pretty boat, pretty fan* NIGHTNIGHT [2] *nightnight office, nightnight boat* HI [2] *hi plane, hi mommy* MORE [2] *more taxi, more melon*	IT [5] *do it, push it, close it, buzz it, move it*	20 unclassified combinations (e.g., *mommy sleep, milk cup, oh my see*)
**Andrew (age 19–23 months)**		
ALL [12] *all broke, all buttoned, all dry, …* MORE [11] *more cookie, more hot, more read, …* NO [10] *no bed, no home, no fix, …* OTHER [10] *other bib, other pants, other piece* I [3] *I see, I shut, I sit* SEE [3] *see baby, see pretty, see train* HI [3] *hi Calico, hi mama, hi papa* COME [2] *mail come, mama come*	OFF [6] *boot off, light off, water off* BY [2] *airplane by, siren by* PREPOSITION THERE [11] (e.g., *clock on there, milk in there, light up there*)	20 unclassified combinations (e.g., *all done milk, byebye back, off bib*)
**Stephen (age 23–24 months)**		
WANT [16] *want baby, want do, want up*, … IT [14] *it ball, it daddy, it fall* … THERE [11] *there ball, there doggie, there byebye car* …THAT [5] *that box, that Dennis, that doll* SEE [4] *see ball, see doll, see record, see Stevie* HERE [4] *here bed, here checker, here doll, here truck* MORE [2] *more ball, more book* BEEPPEEP [2] *beeppeep bang, beeppeep car* WHOA [2] *whoa cards, whoa jeep*	DO [4] *bunny do, daddy do, momma do, want do*	16 unclassified combinations (e.g., *bunny do sleep, pon baby, Betty byebye car*)

*Pivots are shown in capital letters, followed by the number of occurrences in square brackets in the files listed for each child, and examples of the Pivot-Open or Open-Pivot structures from the child's speech*.

It is possible to interpret Braine's data in terms of the earliest occurrences of Merge. The children in Braine's study combined two words together, and moreover these children favored the Pivot-Open pattern (although Open-before-Pivot did occur; see the next section), consistent with the children having adumbrated, if not mastered, the Head—Complement/Modifier pattern of English.

### Is Headedness Immediately Evident?^2^

Although Braine's evidence favors the order Pivot-before-Open, these are not the only orders that occur. The opposite order (Open-before-Pivot in Braine's analysis) is also found, as shown by the data with *it* in Braine's data for Gregory; *off, by, come and P-there* for Andrew; and *do* for Steven. More recent research has shown that in early stages word order can be variable: strings that must be interpreted as Subject—Verb, Verb—Subject, Object—Verb and Verb—Object are attested in languages with SVO order (Tsimpli, 1992 [quoted in Galasso, 2001], Galasso, 2001). Thus, it may be the case that at a very early stage the child combines two words without attention to headedness. Nonetheless, the evidence favors the rapid development of a system in which asymmetric Merge is found in child language^3^.

This conclusion is supported by the only study we are aware of a language that is head-final. Jordens et al. (2008) examined the development of one child speaking Japanese, Jun, and found indeed that there was a pattern that can be interpreted as Open-Pivot^4^. Jordens et al. report that in his utterances Jun used a pattern in which the utterance final position was occupied by a particle, as in (1),


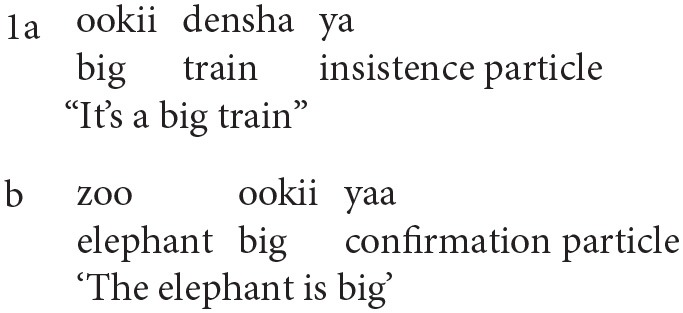


We can take such utterances as a realization of the pattern Open-Pivot, with the particle serving as the pivot. The context makes clear that the child is not simply mimicking the adult('s) speech; the child latches onto a pattern with an utterance final particle as Pivot despite the absence of such a particle in the immediate speech context. Jordens et al. observe that at an early age (the files when Jun is 1;11) the order we are interpreting as Open-Pivot accounts for half the analysable utterances (the remaining half mostly consists of one word utterances when Jun is engaged in naming pictures, objects, etc.). And so, the evidence suggests that the order Pivot-Open is preferred in English, but Open-Pivot is preferred (for the admittedly small amount of data) in Japanese, in accord with the branching pattern of the language being learned.

### Some Issues With Data Interpretation

Assuming that headedness is present, one question that arises with respect to the data is what the labels associated with the heads are. Braine (1963) observes that Gregory appears to adumbrate a Noun/Adjective vs. Verb distinction. It is mainly nouns and adjectives that serve as Open words in the Pivot-Open order, and only verbs that serve as Open words in Open-Pivot order. Tentatively, we can assume a progression from an unspecified head to categories that resemble the specific categories of English:


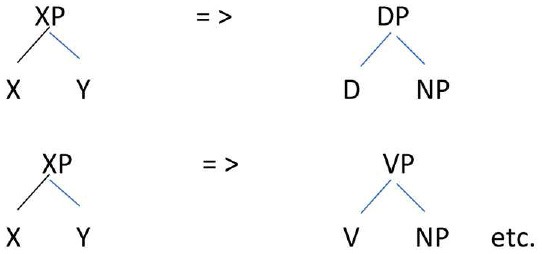


Such a progression does not imply that the categories all at once switch from a general category label to specific categories. The development may be dependent on the lexical categories merged and may be piecemeal. For example, the child Gregory in Table 1 may have a nascent category VP in his utterances with the final pivot *it*, and a nascent category AP in his utterances with the initial pivots *big* and *pretty*.

The development takes place within two or three months in the second year, and may vary from child to child. The data from Allison in Table 2 shows a fairly clear breaking point between 22 and 24 months. At 22 months she produces almost no utterances longer than two words and at 24 months she is capable of producing an utterance of six words. However, Allison also shows typically telegraphic speech, with almost no articles or prepositions, as the examples of her utterances illustrate. The data from Abigail in Table 2 shows that at more or less the same age as Allison and the three children in Braine's data she has already plausibly developed a rich repertoire that enables significant sentence complexity. At 24 months, she produces sentences with auxiliary verbs, including the sentence with the (presumably epistemic) modal *must*: *Mummy must have gone shopping*.

**Table 2:** Analysis of the speech from Allison (MacWhinney, 2000; Bloom 1973 corpora files 1–4) and Abigail (MacWhinney, 2000, Wells corpora files 1, 2, and 3).

**Utterance length**	**1-word**	**2-word**	**3-word**	**4-word**	**5-word**	**6-word**
**ALLISON**						
Example(s)	*Wiping; chair; eat*	*Baby eat; mommy open; blouse on*	*Baby down chair; baby eat cookies; eat apple juice*	*Put away Allison bag; help cow in table; drink apple juice again*	*Drink apple juice right here*	*Sit down right here next truck*
File 1, 16 months (1;04.21)	347	39	7^1^			
File 2, 19 months (1;07.14)	345	11		1^2^		
File 3, 20 months (1;08.21)	375	49	2			
File 4, 22 months (1;10,00)	154	81	28	5	1	1
**ABIGAIL**						
Example(s)	*bike; writing; mummy*	*a bang; this way; baba mummy?*	*I want mummy; this cut it; goes on there*	*do it for me; this is a boot [= boat]; the bell ring Mummy*	*Mummy must have gone shopping*	
File 1, 18 months (1;05.28)	30	11	2	1		
File 2, 21 months (1;08.27)	29	22	6	4		
File 3, 24 months (2;00.01)	41	26	16	10	1	

1 *All involve [wi(deh)]. [wi(deh)] is a sequence of sounds that occurs in Allison's speech which have no identifiable referent*.

2 *Repetition of mother's utterance*.

It has been more or less a given assumption in child language studies that the one word stage is followed by a two word stage, but that there is no separable three word stage. This is broadly consistent with the data from Allison in Table 2, and would follow from a picture of development in which the child first “practices” with two word utterances (the output of simple Merge) and subsequently commands the operation of Merge sufficiently well for several applications of it to occur in a single utterance. Consistent with this, Braine reports an increase in utterance length at around the fifth and sixth months of his study. However, he does not specify the proportions of two vs. three and more word utterances before and after the upsurge.

We examined some CHILDES files and found that utterances with pivots are not as frequent as we might have expected on the basis of Braine's data, although they are not completely absent. For example, in the file for the child Eric at 1;10 (MacWhinney, 2000, Bloom 1970 files) Eric has a pivot utterance *no more X*, which accounts for 18 out of 21 three word utterances. Overall, it is possible that the diary method used by Braine may be more revealing of stages than the method of short recordings that characterizes the CHILDES files.

### Bare Phrase Structure

To what extent is (asymmetric) Merge superior to the traditional X-bar theory of phrase structure in explaining children's behavior? Chomsky (1995, pp. 241–249) sketches the “bare phrase structure” theory, of which Merge is the essential component, and compares the bare phrase structure approach to X-bar theory. Chomsky proposes that X-bar structures along the lines of (2a) be replaced by (2b) (his 8a and 8b),


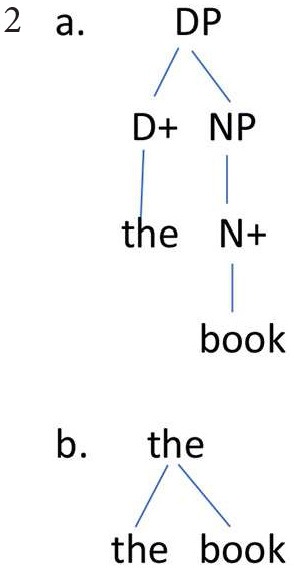


The components of (2b), *the* and *book*, are abbreviations for the set of features in the lexicon that make up those words. We have described a child's development as a progression from random (unordered) conjunctions to the headed combinations of asymmetric Merge. This development will surely take account of frequency in the input of various structures. The child does not need to construct intermediate layers of representation of X-bar theory unless the input motivates these layers. Thus, the acquisition of bare phrase structure can be argued to provide a simpler account than X-bar theory of the move from fixed and limited combinations to grammatically licit productive combinations that result from asymmetric Merge.

### Summary

What are the advantages of the analysis of phrase structure in terms of the operation of Merge? As stated in the previous section, the Merge account obviates the need for intermediate layers of structure. Other than that, the Merge account of language development must be blended with an account that includes properties of the perceptual interface in a Minimalist model. In the Minimalist framework, the interfaces between syntactic representation and the sensory-motor system (phonetic form) and conceptual-intentional system (logical form) are constrained by extra-linguistic factors (Hauser et al., 2002), including cognitive structures, pragmatics and memory limitations. In order to use asymmetric Merge, the child must take onboard distributional evidence from the language s/he is learning, and an individual child may differ in the rapidity with which he or she moves from the simple operation of Merge to the capacity to execute multiple cases of Merge in a single utterance, as illustrated by the contrast in behaviors between Allison and Abigail. The change from X-bar theory to Merge does not in any obvious way change the puzzle of what the connection is between the evidence of the child's perceptions and his or her construction of a grammar, although the recognition of the role of interface conditions in the Minimalist model provides a framework for exploration.

## More on Interface Constraints

In this section, we look at another area of grammar in which we argue that interface constraints are needed for a full explanation of language development.

### The Interpretation of Pronouns and the Organization of the Processor

Reuland (2001) develops a minimalist alternative to the Binding Theory of Chomsky (1981), building on earlier work by Reinhart and Reuland (1993). In Reuland's analysis, principles A and B (governing the distribution of reflexive pronouns and definite pronouns, respectively) are replaced by a requirement that verbs are interpreted reflexively only when they are combined with a reflexive pronoun, i.e., verbs that are interpreted reflexively do not permit a definite pronoun with a reflexive interpretation. This excludes a sentence such as (3) from having an interpretation in which *de man* and *hem* corefer.


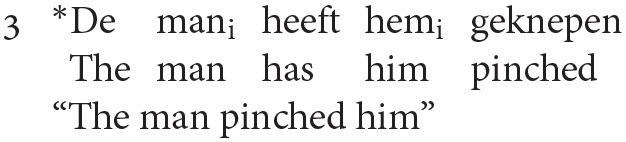


A separate analysis is required by Reuland to exclude co-reference in exceptional case marking (ECM) contexts, such as (4). This is achieved by a condition on A-chains (chains formed between arguments) requiring that at most one member of the chain (the head) is marked as +R(eferential), where +R items are those that carry full specification of φ-features and case. Since both pronouns and lexical NPs are +R, (4) is ungrammatical.


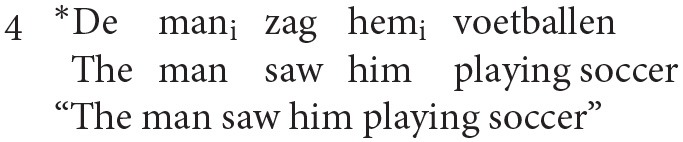


Reuland draws a distinction between the levels of grammar required to determine the ungrammaticality of (3) vs. (4). The ungrammaticality of (3) can be determined in the narrow syntax, by virtue of the requirement that a predicate, if interpreted reflexively, excludes a definite pronoun from its domain. (3) is also ungrammatical because it violates the condition on A-chains. The ungrammaticality of (4) relies solely on the condition on A-chains, which in turn mandates access to discourse structure. Reuland argues that the degree of crosstalk between levels of representation determines the complexity of a sentence: the ungrammaticality of (3) can be determined by reference to the narrow syntax requirement on reflexive predicates, whereas the ungrammaticality of (4) is determined by the narrow syntax rule governing the well formedness of chains, which in turn requires access to a discourse related phenomenon (the referentially of the pronoun).

Ruigendijk et al. (2011) provide striking evidence that Dutch, Spanish and Italian children aged 4–5, draw a distinction between sentence types (3) and (4) in their native languages, with many more errors in the case of sentence type (4). Ruigendijk et al. take this as an indication that Reuland's analysis is correct, in contrast to the analysis of Chomsky (1981), which treats both (3) and (4) as violations of principle B of the binding theory (in which the domain for computing reference of pronouns was the whole sentence in both 3 and 4). See also Brunetto (2012) for further experiments on ECM constructions in child Italian.

Ruigendijk et al. propose that the ungrammaticality of (4) is known to children, but that a lack of processing resources intervenes to produce errors on that sentence type. Part of the evidence they cite is a study by Sekerina et al. (2004) on the processing of sentences such as (5), in which both a pronoun and a reflexive are acceptable with reference to *the boy*.


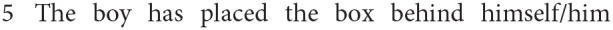


Sekerina et al. found that children aged 4–7 years as well as adults were aware of both, in a task in which the participants had to choose between two pictures, one representing the internal reading of the reflexive/pronoun (i.e. the boy with the box behind his own back) and the other representing the external reading (i.e. the boy with the box behind the man's back), while their fixations on each picture were recorded. After a period in which both internal and external fixations were about equal, both child and adult groups fixated on the picture representing the sentence internal reading more than on the picture representing the sentence external reading. However, the children took longer to establish the pattern of fewer fixations for pronouns. When asked to choose one of the two pictures, the adults chose the picture representing the sentence external reading in about one fifth of their responses to pictures with pronouns, whereas the children almost never chose the picture representing the sentence external reading. Thus children in this study showed awareness of the grammaticality of the pronoun as well as the reflexive in sentences such as (5), as evidenced by their fixation pattern, but failed to reflect that awareness in a more resource-intensive picture pointing task, in which they consistently chose the internal reading^5^.

A widely accepted (but far from uncontroversial) model of sentence processing places access to discourse representations toward the end of the chain of operations in comprehending a sentence (see for an early example of such a model, Forster, 1979). Thus, we can see a parallel between Reuland's analysis and a processing model. If operations that are at the end of the comprehension sequence are less efficiently executed (for reasons of, for example, lower working memory), then we have the potential to explain why children do worse on sentences such as (4) than they do on sentences such as (3).

### Is the Minimalist Program an Advance on the Government and Binding Model?

Notice, however, that the parallelism is not exact between Reuland's analysis and a processing model which entails that sentence-external reference is less easily accomplished than sentence-internal reference. A Minimalist-friendly processing model would not only provide an explanation of the pattern of findings with respect to Principle B summarized in the preceding section, but also be extended to other results with Principle C of the binding theory and the interpretation of control structures described in the following paragraphs.

In an act-out experiment, Goodluck and Solan (2001) required 3–6 year old French-speaking children to act out to sentences such as (6–7),


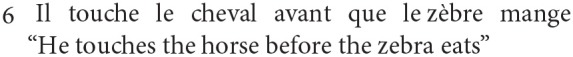



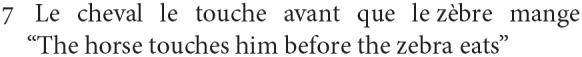


Principle C of the Chomsky's binding theory blocks coreference between *Il* and *le zèbre* in (6), since *il* c-commands *le zèbre*. In (7), however, coreference is possible between the pronoun *le* and *le zèbre*, since the pronoun is contained within the main clause VP, and does not c-command the adjunct clause. In acting out sentence type (6), there was a difference between the younger children (3–4s) and the older children (5–6s). The younger children were inclined to act out (6) as if it was (7), whereas the older children were more able to select an unmentioned animal as referent of the pronoun. Thus, the younger children gave a response that was incorrect for the stimulus (but nonetheless corresponded to a grammatical sentence type); they did not go outside the sentence for a referent of the pronoun.

An additional result argues that younger children have problems with accessing material not mentioned in the sentence. Goodluck et al. (2001) studied the acquisition of controlled complements in Spanish. In the adult grammar, the null subject (E[mpty] C[ategory]) of the complement to *quiere* (“want”) obligatorily refers to an unmentioned entity when the complement is subjunctive,


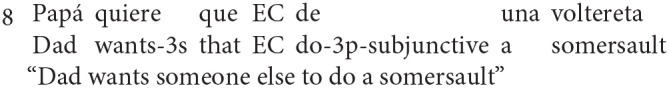


In an experiment in which adults and children acted out sentences with dolls, adults *never* gave a response in which the main clause subject was made co-referential with the EC. Four to five year old children gave such a response in 89% of cases; even by age 6–7, there were 46% of such responses. The younger children failed to take into account the requirement to go outside the sentence in the case of subjunctive complements^6^.

Thus, we have evidence from different areas of grammar (Principles B and C of the binding theory and control) that children slip up when the grammar requires them to look outside the local domain to analyze the input. We need a model that allows for:

- the limitations (individual and particular to groups) in working memory;
- the limitations (perhaps relating to [a]) in span which can be accessed, such as the “sentence/clause bound” properties of responses to (5–7).

The Minimalist program here offers an advantage over the Government and Binding model. By recognizing the need for interface conditions such as working memory capacity, we can provide a unified explanation of the phenomena from different areas of grammar. Concomitantly, there is potentially a reduction of the role of learning in acquisition. For example, Hamann (2011) reviews the extensive literature on the acquisition of the binding theory (Principles A and B), including debates concerning whether pragmatic principles are learned/develop over time to account for the slower mastery of pronouns as opposed to reflexives. By placing the burden on the processing mechanism in explaining children's problems in understanding the grammar of pronouns, we can reduce (but not eliminate) the need for learning. We can reduce it partly by explaining the errors children make as a consequence of the limited span (b, above), but we cannot eliminate the need to learn, for example, the language particular distributions of clitic vs. non-clitic pronouns, which may be affected by their frequency, *inter alia*. Moreover, this allows for a picture in which the hierarchy of operations where narrow syntax takes precedence over operations that involve cross modular specification, such as access to discourse content and non-linguistic context (Grillo, 2008, cited in Hamann, 2011) to be preserved for children, as we would expect if the basic organization of the processor is the same for children as for adults^7^.

One may ask, is pushing the explanation of development in terms of interface conditions an advantageous move? Another example is found in the development of wh-movement. The error of construing a question such as (9), in which lower clause extraction is blocked by the wh-island constraint, with a referent suitable for the lower wh-word (e.g., *Cookies*) has been found in studies of child language, beginning with de Villiers et al. (1990). The studies used a variety of techniques and suggested that the child's grammar was not adult-like at some stage (Thornton, 1990; McDaniel et al., 1995; de Villiers et al., 2011). Slavkov (2015) also found the difficulty with wh-islands for adult second language learners.


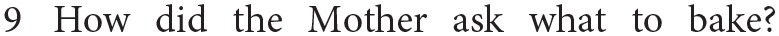


Jakubowicz (2011) outlines a Derivational Complexity Metric, which states that the number of movements involved in the derivation of a sentence determines its difficulty. The error of construing the lower wh-word as an answer to a question such as (9), and the error of producing questions with a medial copy of a wh-word (incorrect for the adult language), can be accounted for under a phase based complexity metric, which starts the computation at the lowest cycle, and founders for lack of processing capacity. Jakubowicz makes appeal to working memory capacity:

“…the number of phases that the wh-phrase needs to go through on its way to the left edge of the matrix CP exceeds the limits of processing resources/working memory capacity” (p. 344)^8^.

Working memory capacity is variable (children and adults differ in their capacities), and the calculation of the number of phases by the performance mechanism yields a potential explanation of children's behavior. This contrasts with an explanation in terms of a non-adult grammar for the child. Parallels with adult languages that permit intermediate copies of a wh-word have been drawn to suggest that the child has a different grammar; see for example, McDaniel et al. (1995). Although it is not clear that Jakubowicz' theory can handle all the data, the advantage of an explanation of children's behavior in terms of interface conditions on working memory is that the theory of language acquisition does not have to account for the unlearning of an incorrect grammar^9^.

## Concluding Remarks

We have suggested that the Minimalist model of bare phrase structure may offer a superior account of the early stages of acquisition of word order than traditional X-bar theory.

The Minimalist model includes interface conditions. The combination of theoretical principles with the mechanisms for producing and understanding sentences can result in a simpler theory of acquisition: the interface condition account of children's behavior reduces the need to posit grammars that must be corrected in the course of acquisition. To the extent that the Minimalist model explicitly recognizes interface conditions, the Minimalist framework is superior to previous frameworks, such as Government-Binding theory.

The examples discussed here are just two of the examples of how children's behaviors might be accounted for in Minimalist terms. Other areas of language development have scarcely begun to be explored from a Minimalist perspective. For example, the rich morphology of some polysynthetic languages such as Inuktitut is learned at a very early age, under 12–14 months (Crago and Allen, 2001), in contrast to the impoverished morphology of languages such as English, which may take until 4 years to be mastered (Brown, 1973). Is it the case that the early mastery of Inuktitut derives from the direct access to material in the numeration (the list of words and morphemes at the beginning of a derivation), without the need for movement operations to match up the morphology with the functional categories that are needed in a language such as English? Or is it the case that the input in languages such as Inuktitut is richer than in English, leading to earlier acquisition? Or do both factors play a role? These questions are unanswered, but offer the promise of a rich future for the Minimalist theory and language acquisition data.

## Data Availability Statement

The Childes data files in Table 2 were analyzed for the study.

## Author Contributions

HG drafted versions of the paper. NK revised the drafts, suggesting revisions and additions.

## Conflict of Interest

The authors declare that the research was conducted in the absence of any commercial or financial relationships that could be construed as a potential conflict of interest.

## Footnotes

^1^Braine used the term “X” for “Open” class words.

^2^Our thanks for a reviewer for his/her comments on the issue of headedness.

^3^See Yang and Roeper (2011, p. 563–464), for a similar point. Yang and Roeper cited in Drozd (2001). Their bibliography includes the reference to Drozd (2001) in our bibliography, but it seems that the reference should be 2002. Yang and Roeper's interpretation in terms of asymmetric Merge differs from Drozd' (2002) own interpretation in terms of a reduction of adult forms, although the two are not incompatible.

^4^Jordens et al. (2008) use the terms “Predicate” and “Link,” respectively.

^5^Ruigendijk et al. also cite several studies that show slowed processing by brain-damaged patients.

^6^Brunetto (2012) writes: “As previous studies on the acquisition of control suggest, it is very unlikely that children's problems in the interpretation of the embedded subject consist in assigning PRO an external referent. These errors, in fact, are very rare already at age 3 and much evidence seems to indicate that subject control is mastered very early (Goodluck et al., 2001)” (p. 190). This seems to us to miss the point. The sentence internal response of Spanish-speaking children is an error. The source of this error is potentially underdetermined. It could be that Spanish children lack the requisite knowledge subjunctive morphology, or that (as we contend) they lack the capacity to go outside the sentence for a referent of the EC. Castilla-Earls et al. (2018) summarize evidence that typically developing Spanish children correctly produce the subjunctive from as young two years.

^7^This is in contrast to the position that Hamann takes: “For explaining acquisition facts, it has to be assumed that the hierarchy is not in place yet. In particular, narrow syntax is not the cheapest option for the child, perhaps because full automatization or the step from the particular to the general is not achieved yet.” (p. 260–261).

^8^The data from Jakubowicz' article is complex and requires additional assumptions to be made; however, the basic point concerning the role of processing limitations holds.

^9^A similar analysis to that of Jakubowicz' is also made by de Villiers et al. (2011); de Villiers et al. suggest that a phase-based movement of a lower wh-word to the semantic/interpretative component may lead to errors such as that found for sentences such as (9).
